# BioJava 5: A community driven open-source bioinformatics library

**DOI:** 10.1371/journal.pcbi.1006791

**Published:** 2019-02-08

**Authors:** Aleix Lafita, Spencer Bliven, Andreas Prlić, Dmytro Guzenko, Peter W. Rose, Anthony Bradley, Paolo Pavan, Douglas Myers-Turnbull, Yana Valasatava, Michael Heuer, Matt Larson, Stephen K. Burley, Jose M. Duarte

**Affiliations:** 1 European Molecular Biology Laboratory, European Bioinformatics Institute, Wellcome Genome Campus, Hinxton, Cambridge, CB10 1SD, UK; 2 Zurich University of Applied Sciences (ZHAW), Zurich CH-8021, Switzerland; 3 San Diego Supercomputer Center, UCSD, San Diego, CA 92093, USA; 4 Research Collaboratory for Structural Bioinformatics Protein Data Bank, San Diego Supercomputer Center, University of California, San Diego, La Jolla, CA 92093, USA; 5 Structural Bioinformatics Laboratory, San Diego Supercomputer Center, UCSD, San Diego, CA 92093, USA; 6 Structural Genomics Consortium, University of Oxford, Oxford OX3 7DQ, UK; 7 Department of Chemistry, University of Oxford, Oxford OX1 3TA, UK; 8 Diamond Light Source Ltd., Didcot OX11 0DE, UK; 9 Genomnia srl, Bresso, Milan, Italy; 10 Institute for Neurodegenerative Diseases, University of California, San Francisco, CA 94143, USA; 11 RISELab, University of California Berkeley, Berkeley, CA, USA; 12 DNASTAR, Madison, WI, USA; 13 Research Collaboratory for Structural Bioinformatics Protein Data Bank, Institute for Quantitative Biomedicine, Rutgers, The State University of New Jersey, Piscataway, NJ 08854, USA; 14 Rutgers Cancer Institute of New Jersey, Robert Wood Johnson Medical School, New Brunswick, NJ 08903, USA; Hebrew University of Jerusalem, ISRAEL

## Abstract

BioJava is an open-source project that provides a Java library for processing biological data. The project aims to simplify bioinformatic analyses by implementing parsers, data structures, and algorithms for common tasks in genomics, structural biology, ontologies, phylogenetics, and more. Since 2012, we have released two major versions of the library (4 and 5) that include many new features to tackle challenges with increasingly complex macromolecular structure data. BioJava requires Java 8 or higher and is freely available under the LGPL 2.1 license. The project is hosted on GitHub at https://github.com/biojava/biojava. More information and documentation can be found online on the BioJava website (http://www.biojava.org) and tutorial (https://github.com/biojava/biojava-tutorial). All inquiries should be directed to the GitHub page or the BioJava mailing list (http://lists.open-bio.org/mailman/listinfo/biojava-l).

This is a *PLOS Computational Biology* Software paper.

## Introduction

BioJava was launched in 2000 as an open-source Java library for bioinformatics focused on biological sequences and alignments [1]. The functionality of the library has grown over the years, ranging from parsers for common biological file formats to state-of-the-art tools for sequence and structural comparisons [2]. Following the major rewrite of the code base in version 3, the library consists of eleven independent modules that provide access to biological sequences, structures and common bioinformatics routines [3]. In addition to mature data structures for sequence analysis, recent work has yielded an expansion in features for analyzing macromolecular structure data. BioJava has also adopted best practices in software engineering, including continuous integration, unit testing, and code review. Adherence to these practices makes BioJava suitable for inclusion in major bioinformatics pipelines, databases and software.

Bioinformatics is an open and collaborative field, as demonstrated by the many Bio* projects that exist for different programming languages. BioJava is a popular option for method and software development thanks to the tooling available for Java and its cross-platform portability. Other popular projects like BioPerl [4] and BioPython [5] offer great scripting flexibility, now also available in the Java world via the JVM-based scripting languages. BioJava consists of a central code repository, while other projects like R/Bioconductor [6] are decentralized collections of packages developed and maintained independently. The popularity and usability of the Bio* projects is closely tied to the programming language, and therefore in constant evolution.

At present, BioJava is a well-established project and continues to be actively maintained by a diverse user and developer community. The library has accepted contributions from 65 different developers since 2009, accumulated 224 forks and 270 stars on GitHub, and BioJava binaries were downloaded more than 19 thousand times over the last year. The BioJava project is also supported by the Open Bioinformatics Foundation (https://www.open-bio.org), a non-profit group dedicated to promoting the practice and philosophy of Open Source software development and Open Science within the biological research community.

## Design and implementation

### The BioJava modules

The BioJava library is organized into several modules for maximum flexibility. Users can choose what subset of modules to depend on in their projects.

The core module provides interfaces and routines to work with protein and nucleotide sequences. Some of the functionality includes parsing sequences from local files and remote resources, conversion between file formats and gene to protein translation. This module acts as a base module and others can depend on it. The alignment module supplies standard algorithms and data structures for pairwise and multiple sequence alignments. In version 5, the phylo module was integrated into the alignment module to support phylogenetic analyses using the Forester library [7] (https://github.com/cmzmasek/forester). The structure module provides data structures and algorithms to parse, manipulate and compare 3D structures of biological macromolecules, and the structure-gui module allows visualization of structures and structure alignments in Jmol [8].

Other smaller modules provide more specific functionality for different Bioinformatics fields. The genome module deals with genomic data supporting memory-efficient parsers for GTF, GFF2, GFF3 and FASTQ file formats. For protein analyses, the aa-prop module provides a range of physicochemical properties (e.g. molecular weight, isoelectric point, extinction coefficient, net charge), the protein-disorder module implements a parallelized version of the Regional Order Neural Network (RONN) [9] for disorder prediction, and the modfinder module implements routines to identify protein modifications observed in 3D structures [10]. Survival analyses using the Kaplan-Meier estimator [11] are possible with the survival module. The ontology module adds support for ontologies and parsing OBO files. And finally, several bioinformatics services can be accessed using REST protocols using the ws module.

More detailed information about each BioJava module can be found in the Wikipedia page (https://en.wikipedia.org/wiki/BioJava) and BioJava documentation, as well as in the previous publication [3].

### New features

A number of new features have been added to BioJava in the last few years, most of which are related to structural biology data handling. Below we highlight a few of the most relevant.

#### Updated structure data model

BioJava uses a hierarchical data model to represent biological structures. In version 5, the representation has been adapted to closely follow the data model defined in the PDBx/mmCIF dictionary. Instances of molecular entities (chains) are separated into 2 types: polymeric and non-polymeric chains, facilitating the traversal of the data and explicitly separating small molecules (ligands, cofactors, ions, etc.) from polypeptides and nucleic acid chains.

#### New structure file formats

The structure module now supports reading, writing, and fetching structures in PDB, PDBx/mmCIF, and MMTF formats, thereby enabling representation of very large structures, support for rich annotations, and conversion between formats. Inclusion of the MacroMolecular Transmission Format (MMTF) [12, 13] has also led to performance improvements compared to the other data formats.

#### Multiple structural alignments

**Fig 1:**
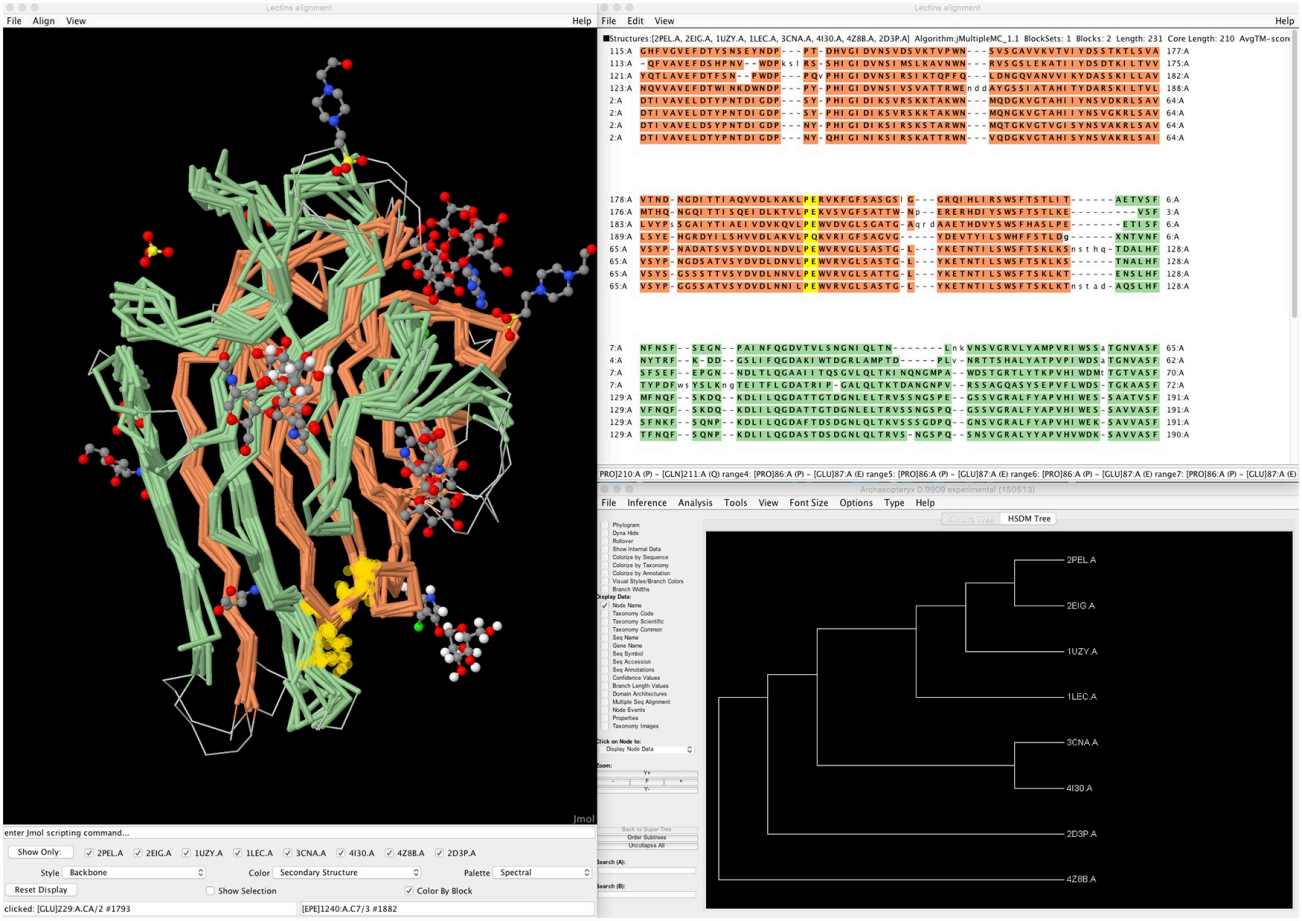
Multiple structure alignment of circularly permuted lectins generated and visualized with BioJava. Implementations of CE-CP and CE-MC were used for the structural alignment, visualized using the Jmol based structure panel (left), the multiple alignment panel (top right), and a Forester based dendrogram of structural similarities (bottom right).

BioJava implements a wide range of pairwise structure alignment algorithms to perform rigid, flexible, and non-topological alignments. We introduced a custom implementation of the CE-MC procedure [14] in the org.biojava.nbio.structure.align.multiple package to generate multiple structure alignments by combining the output of pairwise alignment algorithms. Results are stored in a novel hierarchical data structure that supports rigid, flexible, and non-topological multiple structure alignments. Tools to manage and visualize alignments have also been adapted to enable multiple aligned structures, as demonstrated in Fig 1. More information can be found in the BioJava tutorial.

#### Support for protein assemblies

BioJava provides extensive functionality for working with macromolecular assemblies. Protein complexes can be efficiently aligned using the QsAlign method [15] in the org.biojava.nbio.structure.align.quaternary package. Global, local and internal (within chains) symmetry can also be detected using the QuatSymmetryDetector and CeSymm [16] methods in org.biojava.nbio.structure.symmetry. Moreover, code for reconstruction of the crystal lattice via space group operators is available in the org.biojava.nbio.xtal package, which allows users to easily calculate all chain-chain contacts in a protein crystal.

#### Contacts

An efficient spatial hashing algorithm now permits rapid computation of networks of contacts within a macromolecule and between two distinct macromolecules. Contacts can be exposed on a per atom pair basis or summarized at the residue pair level.

#### Accessible surface area

An implementation of the rolling ball algorithm by Shrake and Rupley [17] was contributed to the structure module. This functionality enables surface accessibility calculations at any level of the structure hierarchy. Features such as calculation of relative surface area and buried surface area upon complex formation are now supported. For example, the functionality of the popular NACCESS program [18] can be fully mimicked with the available features.

#### Secondary structure

Secondary structure assignments from DSSP [19] can now be parsed from local and remote files or calculated from scratch using a custom implementation of the algorithm in org.biojava.nbio.structure.secstruc. This allows the representation of the 8 possible secondary structure types for any protein structure, even the largest ones in the PDB.

#### Improved genomic parsers

The GenBank parser in org.biojava.nbio.core.sequence.io was improved to allow retrieval of genomic features and support nested locations, following the INSDC specification. In addition, BioJava SearchIO, an extensible system for managing generic genome query results, has been implemented and used to store BLAST search results. The Java Service Provider Interface (SPI) is used to allow the system to be extended at runtime to additional types of search results.

### BioJava release cycle

BioJava releases depend on the number and importance of contributions made to the library. Developer contributions happen via GitHub’s pull requests, where new code and fixes are reviewed by the community.

Since version 4, the semantic versioning philosophy has been strictly followed. Changes that break the API represent new major releases, additions to the API are minor releases, and bug fixes are regarded as bugfix releases. We have since released two major versions of the library (version 4 in January 2015 and version 5 in March 2018), and two minor releases for BioJava 4 and one for BioJava 5. In addition, we routinely released bugfix versions every few months. In December 2018, the library is at version 5.1.1.

All BioJava releases can be found on GitHub (https://github.com/biojava/biojava/releases). In addition, a manually curated changelog is kept in a separate file to facilitate understanding of the project’s history.

## Results

**Fig 2:**
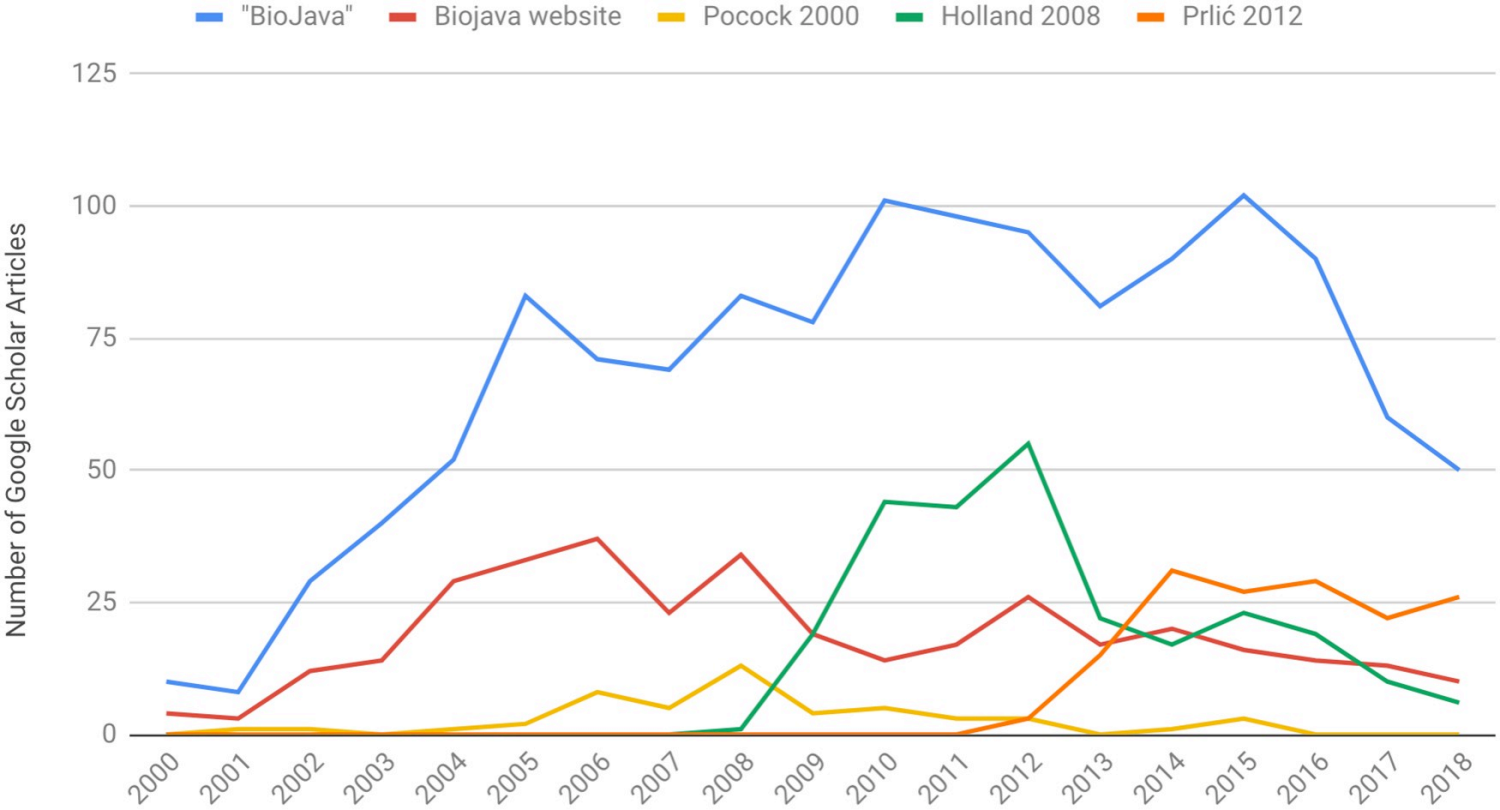
Yearly count of the number of articles that mention the BioJava project (“Biojava”), the Biojava website, or cite the BioJava publications (Pocock 2000 [1], Holland 2008 [2] and Prlić 2012 [3]). Data collected in December 2018 from Google Scholar (https://scholar.google.com).

Throughout its history, the BioJava library has been widely adopted in the scientific community, as demonstrated by the number of BioJava mentions and citations in scientific publications (Fig 2). BioJava is a general purpose bioinformatics library, so it can be used in a broad range of research projects. Examples in the literature include scripting for biological data analysis, the development of novel computational methods, and the creation of integration platforms and web servers for bioinformatics applications. In addition, the open philosophy of the project enhances collaboration between developers, so that many users of the library have eventually contributed back and become developers.

### BioJava for method development

The extensive support of BioJava in basic operations like parsing and manipulating sequences and structures allows developers of novel algorithms to focus all their efforts on the bioinformatics problem itself. For example, BioJava has recently been part of the development of altORFev [20], a method to predict alternative open reading frames in eukaryotic mRNAs, CE-Symm [16], a detector of internal symmetry in protein structures, and EPPIC [21], a predictor of biological assemblies in crystal structures. Similarly, BioJava’s features to generate compressed files and convert between the structural formats were used for the development of the macromolecular compression format (MMTF) [12, 13].

### Integration into large-scale analyses

BioJava can also be used for large-scale bioinformatics applications. Methods of the library can be efficiently run in parallel on large datasets with MMTF-Spark (https://github.com/sbl-sdsc/mmtf-spark), a project to promote scalable analysis of big data in structural bioinformatics. BioJava is also used by CloudPhylo [22], a tool written in Scala and built on Spark that is capable of processing large-scale genomic datasets for phylogeny reconstruction. As another example, BioJava methods were used to compare thousands of protein assembly models to experimental structures during the assessment of biological assemblies in CASP12 [23].

### Scripting and notebooks

In recent years the JVM platform has grown beyond the Java language itself. A plethora of scripting languages that can interoperate with Java libraries have appeared, e.g. Scala, Kotlin, Clojure or Groovy. As a JVM-based library, BioJava can be seamlessly integrated into software written in any of those languages, and a few examples can already be found in the literature. For instance, some work towards Scala integration was accomplished in the BioScala project (https://github.com/bioscala/bioscala). Equally, integration with popular notebook software like Jupyter has become possible through projects like BeakerX (http://beakerx.com) that provide JVM support for Jupyter. In this context, a demo application providing a geometrical analysis of PDB data based on BioJava code is available at https://github.com/sbl-sdsc/biojava-notebooks.

### Academic and commercial software

Last but not least, BioJava is a popular choice for the development of software platforms and web services that integrate several different bioinformatics applications. These include RobiNA [24], an integrated software solution for RNA-Seq-based transcriptomics, the HDX Workbench [25], an integrated desktop program for Hydrogen/Deuterium exchange mass spectrometry (HDX-MS) analysis, and G2S [26], a web-service for annotating genomic variants on 3D protein structures, to mention a few of the many examples. BioJava is also widely used by the RCSB Protein Data Bank (PDB) for their web-services [27], including protein quaternary symmetry annotation and visualization, structural comparisons and the exploration of protein modifications. Finally, the BioJava project powers a number of commercial software products such as those from Genomnia and DNASTAR.

## Availability and future directions

BioJava is an open-source project driven by the community. The library is currently hosted on GitHub, a platform that has simplified project management and enabled best practices in software engineering. Binaries and source code are distributed freely under the Lesser GPL (LGPL) 2.1 license at https://github.com/biojava/biojava.

BioJava 5 is a mature library with extensive support for a wide range of bioinformatics applications. Work in recent years has been focused on tackling challenges with complex structural bioinformatics data. In the coming years, effort will continue to improve usability and stability, whilst reaching into new types of data from new experimental methods and growing bioinformatics fields like genomics and the integration into scientific workflows. The Open Source philosophy will remain central to BioJava, as the project was founded on the firm belief that transparency promotes reproducible science, faster development through scientific and technical contributions by the community, and more robust and better documented code.
